# Emergency care with lay responders in underserved populations: a systematic review

**DOI:** 10.2471/BLT.20.270249

**Published:** 2021-04-29

**Authors:** Aaron M Orkin, Jeyasakthi Venugopal, Jeffrey D Curran, Melanie K Fortune, Allison McArthur, Emma Mew, Stephen D Ritchie, Ian R Drennan, Adam Exley, Rachel Jamieson, David E Johnson, Andrew MacPherson, Alexandra Martiniuk, Neil McDonald, Maxwell Osei-Ampofo, Pete Wegier, Stijn Van de Velde, David VanderBurgh

**Affiliations:** a Department of Family and Community Medicine, University of Toronto, 155 College St, Toronto, ON M5T 3M7, Canada.; b Dalla Lana School of Public Health, University of Toronto, Toronto, Canada.; c Department of Medicine, McMaster University, Hamilton, Canada.; d Division of Clinical Sciences, Northern Ontario School of Medicine, Timmins, Canada.; e Ontario Public Health Libraries Association, Toronto, Canada.; f Laurentian University, Sudbury, Canada.; g Sunnybrook Research Institute, Sunnybrook Health Sciences Centre, Toronto, Canada.; h Division of Clinical Sciences, Northern Ontario School of Medicine, Thunder Bay, Canada.; i Alberta Health Services, Calgary, Canada.; j Wilderness Medical Associates International, Portland, United States of America.; k Department of Emergency Medicine, University of British Columbia, Victoria, Canada.; l Faculty of Medicine School of Public Health, University of Sydney, Sydney, Australia.; m Winnipeg Fire Paramedic Service, Winnipeg, Canada.; n Department of Medicine, Kwame Nkrumah University of Science and Technology, Kumasi, Ghana.; o Humber River Hospital, Toronto, Canada.; p Division for Health Services, Norwegian Institute of Public Health, Oslo, Norway.

## Abstract

**Objective:**

To assess the individual and community health effects of task shifting for emergency care in low-resource settings and underserved populations worldwide.

**Methods:**

We systematically searched 13 databases and additional grey literature for studies published between 1984 and 2019. Eligible studies involved emergency care training for laypeople in underserved or low-resource populations, and any quantitative assessment of effects on the health of individuals or communities. We conducted duplicate assessments of study eligibility, data abstraction and quality. We synthesized findings in narrative and tabular format.

**Findings:**

Of 19 308 papers retrieved, 34 studies met the inclusion criteria from low- and middle-income countries (21 studies) and underserved populations in high-income countries (13 studies). Targeted emergency conditions included trauma, burns, cardiac arrest, opioid poisoning, malaria, paediatric communicable diseases and malnutrition. Trainees included the general public, non-health-care professionals, volunteers and close contacts of at-risk populations, all trained through in-class, peer and multimodal education and public awareness campaigns. Important clinical and policy outcomes included improvements in community capacity to manage emergencies (14 studies), patient outcomes (13 studies) and community health (seven studies). While substantial effects were observed for programmes to address paediatric malaria, trauma and opioid poisoning, most studies reported modest effect sizes and two reported null results. Most studies were of weak (24 studies) or moderate quality (nine studies).

**Conclusion:**

First aid education and task shifting to laypeople for emergency care may reduce patient morbidity and mortality and build community capacity to manage health emergencies for a variety of emergency conditions in underserved and low-resource settings.

## Résumé

**Objectif:**

Évaluer l'impact, sur la santé individuelle et collective, du transfert des interventions de premiers secours dans les endroits disposant de ressources limitées et au sein des populations défavorisées à travers le monde.

**Méthodes:**

Nous avons analysé systématiquement 13 bases de données ainsi que toute littérature grise complémentaire pour y trouver des études publiées entre 1984 et 2019. Les études retenues devaient faire mention d'une formation aux premiers secours pour les non-professionnels au sein des populations défavorisées ou dotées de peu de ressources, mais aussi d'une évaluation quantitative de l'impact sur la santé individuelle et collective. Nous avons dupliqué les appréciations d'admissibilité de l'étude, de qualité et d'abstraction des données. Enfin, nous avons synthétisé les résultats sous forme de textes et de tableaux.

**Résultats:**

Sur 19 308 articles récupérés, 34 études correspondaient aux critères d'inclusion propres aux pays à faibles et moyens revenus (21 études) et aux populations défavorisées dans les pays à hauts revenus (13 études). Plusieurs situations d'urgence étaient ciblées: traumatismes, brûlures, arrêts cardiaques, intoxications aux opiacés, malaria, maladies infantiles contagieuses et malnutrition. Les stagiaires étaient des individus issus du grand public, des non-professionnels de la santé, des bénévoles et des contacts proches de populations à risque, tous formés dans le cadre de cours multimodaux organisés par des pairs et de campagnes de sensibilisation de l'opinion publique. Diverses retombées politiques et cliniques d'envergure ont été constatées: amélioration de la capacité de gestion des urgences dans les communautés (14 études), conséquences positives pour les patients (13 études) et santé collective (7 études). Bien que des effets non négligeables aient été observés pour les programmes de lutte contre la malaria infantile, les traumatismes et l'intoxication aux opiacés, la plupart des études n'ont remarqué que des effets d'ampleur modeste et deux ont rapporté un bénéfice nul. En outre, la majorité d'entre elles se sont révélées de piètre qualité (24 études) ou de qualité moyenne (9 études).

**Conclusion:**

La formation aux premiers secours et le transfert des interventions aux non-professionnels peut contribuer à diminuer la morbidité et la mortalité des patients, mais aussi à développer les capacités communautaires de gestion des urgences sanitaires pour une série de situations dans les milieux défavorisés ou manquant de ressources.

## Resumen

**Objetivo:**

Evaluar los efectos en la salud individual y comunitaria del cambio de tareas para la atención de emergencia en entornos con bajos recursos y poblaciones desatendidas a nivel mundial.

**Métodos:**

Se realizaron búsquedas sistemáticas en 13 bases de datos y en la literatura gris adicional de estudios publicados entre 1984 y 2019. Los estudios elegibles involucraron la formación en atención de emergencia para personas no profesionales en poblaciones subatendidas o de bajos recursos, y cualquier evaluación cuantitativa de los efectos en la salud de los individuos o las comunidades. Se realizaron evaluaciones duplicadas de la elegibilidad de los estudios, la abstracción de datos y la calidad. Se sintetizaron los resultados en formato narrativo y tabular.

**Resultados:**

De los 19.308 documentos recuperados, 34 estudios cumplían los criterios de inclusión de países con ingresos bajos y medios (21 estudios) y de poblaciones desatendidas de países con ingresos altos (13 estudios). Las condiciones de emergencia a las que se dirigían incluían traumatismos, quemaduras, paros cardíacos, intoxicación por opioides, malaria, enfermedades pediátricas transmisibles y desnutrición. Entre los alumnos se encontraban el público en general, los profesionales no sanitarios, los voluntarios y los contactos cercanos de las poblaciones de riesgo, todos ellos formados a través de campañas de educación y concienciación pública presenciales, entre compañeros y multimodales. Los resultados clínicos y políticos más importantes fueron la mejora de la capacidad de la comunidad para gestionar emergencias (14 estudios), los resultados de los pacientes (13 estudios) y la salud de la comunidad (7 estudios). Aunque se observaron efectos sustanciales en los programas para abordar la malaria pediátrica, los traumatismos y la intoxicación por opioides, la mayoría de los estudios informaron de tamaños de efecto modestos y dos informaron de resultados nulos. La mayoría de los estudios fueron de calidad débil (24 estudios) o moderada (9 estudios).

**Conclusión:**

La formación de personas en primeros auxilios y la transferencia de tareas a los legos para la atención de emergencias pueden reducir la morbilidad y la mortalidad de los pacientes y fomentar la capacidad de la comunidad para gestionar las emergencias sanitarias en una variedad de condiciones de emergencia en entornos desatendidos y de bajos recursos.

## ملخص

الغرض تقييم الآثار الصحية الفردية والمجتمعية لتحويل المهام للرعاية الطارئة في البيئات منخفضة الموارد، والفئات السكانية المحرومة حول العالم.

الطريقة قمنا بالبحث بشكل منهجي في 13 قاعدة بيانات ومؤلفات رمادية غير رسمية للدراسات المنشورة ما بين عامي 1984 و2019. تضمنت الدراسات الملائمة تدريبًا على رعاية الطوارئ للأشخاص العاديين في المجموعات السكانية المحرومة أو منخفضة الموارد، وأي تقييم كمي للتأثيرات على صحة الأفراد أو المجتمعات. وأجرينا تقييمات مزدوجة لمدى ملاءمة الدراسة، واستخراج البيانات والجودة. وقمنا بتجميع النتائج في صيغة سردية وشكل جداول.

النتائج من بين 19308 ورقة بحثية تم استرجاعها، استوفت 34 دراسة معايير الاشتمال من الدول منخفضة ومتوسطة الدخل (21 دراسة)، والفئات السكانية المحرومة من الخدمات في الدول مرتفعة الدخل (13 دراسة). وشملت حالات الطوارئ المستهدفة الصدمات، والحروق، والسكتة القلبية، والتسمم بالمواد الأفيونية، والملاريا، والأمراض المعدية للأطفال، وسوء التغذية. وكان من بين المتدربين عامة الناس، وأخصائيو الرعاية الصحية، والمتطوعون، وجهات الاتصال المقربة من السكان المعرضين للخطر، وجميعهم مدربون من خلال حملات التوعية العامة والتثقيف داخل الفصول، وعبر الأقران، والوسائط المتعددة. تضمنت النتائج الهامة سواء الإكلينيكية والمتعلقة بالسياسة تحسينات في قدرة المجتمع على إدارة حالات الطوارئ (14 دراسة)، ونتائج المرضى (13 دراسة)، والصحة المجتمعية (7 دراسات). بينما لوحظت تأثيرات ملموسة لبرامج معالجة الملاريا عند الأطفال، والصدمات، والتسمم بالمواد الأفيونية، إلا أن معظم الدراسات أوضحت أحجام تأثير متواضعة، وأشارت دراستان إلى نتائج غير موثوقة. كانت معظم الدراسات ذات جودة ضعيفة (24 دراسة)، أو جودة متوسطة (تسع دراسات).

الاستنتاج إن تعليم الإسعافات الأولية، وتحويل المهام إلى الأشخاص العاديين للرعاية الطارئة، قد يؤدي إلى تقليل معدلات الإصابة بالأمراض ووفيات المرضى، وبناء قدرة المجتمع على إدارة حالات الطوارئ الصحية لمجموعة متنوعة من حالات الطوارئ في البيئات المحرومة من الخدمات وقليلة الموارد.

## 摘要

**目的:**

旨在评估在世界范围内医疗资源匮乏和服务不足的人群中,紧急护理任务转移对个人和社区健康的影响。

**方法:**

我们系统地检索了 13 个数据库和额外的灰色文献,以查找 1984 年至 2019 年之间发表的研究。符合条件的研究包括在医疗资源匮乏或服务不足的人群中对非专业人员开展的紧急护理培训,以及对个人或社区健康影响的任何定量评估。我们对研究的资格、数据提取和质量进行了重复评估。我们以叙述和表格的形式汇总了研究结果。

**结果:**

在检索到的 19,308 篇论文中,其中 34 项研究符合中低收入国家(21 项研究)和高收入国家医疗资源匮乏人群(13 项研究)的纳入标准。研究针对的紧急情况包括创伤、烧伤、心脏骤停、类阿片中毒、疟疾、儿科传染病和营养不良。接受培训的人员包括普通群众、非医疗专业人员、志愿者和高危人群的密切接触者,所有这些人员都通过课堂教育、同伴互助教育和多模式教育以及公益宣传活动接受了培训。重要的临床和政策结果包括改善社区应对紧急情况的能力(14 项研究)、患者结果(13 项研究)和社区健康(7 项研究)。虽然观察到针对儿科疟疾、创伤和阿片类药物中毒的项目产生了重大影响,但大多数研究报告的效果并不大,而且有两项报告没有结果。大多数研究的质量较差(24 项研究)或一般(9 项研究)。

**结论:**

急救教育和将紧急护理任务移交给非专业人员可以降低患者的发病率和死亡率,并培养社区在医疗资源匮乏和服务不足的环境中应对各种紧急情况的能力。

## Резюме

**Цель:**

Оценить влияние передачи задач по оказанию неотложной помощи в условиях ограниченных ресурсов и недостаточного обслуживания населения на здоровье отдельных лиц и общин во всем мире.

**Методы:**

Авторы провели систематический поиск в 13 базах данных и дополнительно по неиндексированной в базах данных литературе на предмет исследований, опубликованных в период с 1984 по 2019 год. Соответствующие критериям исследования включали обучение неотложной помощи для неспециалистов из групп населения с недостаточным уровнем обслуживания или ресурсов, а также любую количественную оценку воздействия на здоровье отдельных лиц или общин. Проведена двойная оценка пригодности к участию в исследовании, абстракции данных и качества. Результаты обобщены в повествовательной и табличной форме.

**Результаты:**

Из 19 308 отобранных статей 34 исследования соответствовали критериям включения из стран с низким и средним уровнем доходов (21 исследование) и групп населения с недостаточным уровнем обслуживания в странах с высоким уровнем доходов (13 исследований). К числу целевых чрезвычайных ситуаций относятся: травмы, ожоги, остановка сердца, отравление опиоидами, малярия, детские инфекционные заболевания и недоедание. В число стажеров входили представители широкой общественности, специалисты, не занимающиеся вопросами здравоохранения, добровольцы и лица, поддерживающие тесные контакты с населением, входящим в группу риска. Все они прошли обучение в рамках аудиторных, коллегиальных и смешанных образовательных и информационно-просветительских кампаний. К числу важных результатов клинической и политической деятельности относятся: улучшение потенциала общин в области управления чрезвычайными ситуациями (14 исследований), результаты лечения пациентов (13 исследований) и охрана здоровья общин (7 исследований). Хотя в программах по борьбе с детской малярией, травмами и отравлениями опиоидами наблюдались существенные изменения, в большинстве исследований сообщалось о незначительной эффективности, а в двух исследованиях сообщалось о нулевых результатах. Большинство исследований показали низкую (24 исследования) или умеренную (9 исследований) степень эффективности.

**Вывод:**

Просвещение по вопросам оказания первой медицинской помощи и передача функций по оказанию неотложной помощи неспециалистам могут снизить заболеваемость и смертность пациентов и укрепить потенциал общин по управлению чрезвычайными ситуациями в области здравоохранения в различных чрезвычайных ситуациях в условиях недостаточного обслуживания и нехватки ресурсов.

## Introduction

Conditions that could be treated with prehospital and emergency care account for an estimated 24 million lives lost each year in low- and middle-income countries.^1^ Training lay providers and volunteer paramedics to respond to health emergencies is among the most cost-effective health interventions globally, at just United States dollars 6–14 per disability-adjusted life year saved.^2^ In May 2019, the World Health Organization (WHO) resolved to improve emergency care in all Member States, including through informal prehospital systems.^3^

International guidelines define first aid as “initial care provided for an acute illness or injury” or “help to a suddenly ill or injured person which is initiated as soon as possible and continued until that person has recovered or medical care is available”.^4^^,^^5^ Teaching first aid to laypeople is part of a 150-year medical humanitarian tradition and a vital component of both formal and informal prehospital care systems.^6^ Over 15 million people in 52 countries receive education in first aid each year from member organizations of the International Federation of Red Cross and Red Crescent Societies alone.^7^ First aid education enhances bystanders’ helping behaviours in emergencies, but it remains unclear what interventions can be delivered effectively by laypeople to save lives and reduce morbidity.^8^

Laypeople with training in first aid can improve access to care for underserved populations, and often deliver the only emergency health-care services in low-resource settings.^1^ Where lay responders are taught first aid to enhance patients’ access to essential interventions, first aid education is a part of the broader concept of task shifting. WHO defines task shifting as “the rational redistribution of tasks within health workforce teams”, specifically from specialized professionals to providers with less training, lay caregivers and patients.^9^ Task shifting improves access to care and outcomes for maternal and child health, chronic and mental health conditions, and communicable diseases.^10^^–^^13^ Less is known, however, about task shifting in emergency health care.

The purpose of this systematic review was to identify the individual and community health effects of task shifting for emergency care in underserved populations and low-resource settings. We hoped to guide programme developers and policy-makers towards interventions that had been subject to evaluations with demonstrable health effects, and to help researchers and evaluators to understand and optimize these initiatives.

## Methods

We developed, registered and published a systematic review protocol based on the PRISMA (Preferred Reporting Items for Systematic Reviews and Meta-Analyses) Statement (PROSPERO CRD42014009685).^14^ Our methods did not deviate from the protocol. We report our review according to the PRISMA guidelines and Synthesis without Meta-Analysis extension.^15^^,^^16^ Further details can be found in in the authors’ data repository.^17^

### Eligibility criteria

We designed the research question for the review according to the Population, Intervention, Comparator, Outcome, Time (PICOT) framework.^18^ Studies were eligible for inclusion if they were conducted in underserved or low-resource populations with any emergency health condition (P); involved first aid or emergency care training or education for laypeople (I); made a comparison with no training or with any other forms of education (C); conferred any individual or community health benefit for emergency health conditions (O); and were conducted over any duration of time (T).

Box 1 shows the inclusion criteria and terms used to define the populations, interventions and outcomes of studies. Studies were included if they incorporated all of the criteria. We included studies published after 1984 with no language restrictions or other exclusion criteria. We included randomized trials, quasi-experimental and observational studies including case series and before-and-after designs, programme evaluations and quality-improvement studies.^22^

Box 1 **Inclusion criteria and definitions of terms for the systematic review of first aid by lay responders in low-resource settings and underserved populations**

**Population criteria**

Underserved or low-resource population: A group that faces any barrier to accessing organized prehospital emergency medical services, including geographical, financial, occupational, sociopolitical, ethnocultural, infrastructural or informational barriers.^14^ We excluded people serving in the military or populations living in war zones from this definition.

Emergency health condition: Health problem(s) where treatment should occur within minutes or hours to reduce suffering, morbidity or mortality. Task shifting for routine intrapartum and perinatal care has been reviewed systematically elsewhere and we therefore excluded it from our definition of emergency health conditions.^10^^,^^19^^,^^20^

**Intervention criteria**

First aid or prehospital emergency care: Any effort to identify, care for or treat an emergency health condition in a prehospital or out-of-hospital setting. First aid may be definitive care or may involve transition to more advanced care.^4^^,^^5^^,^^21^

Training or education: Any effort intended to confer knowledge or skills to a person, or change their attitudes and behaviours.

Laypeople trainees: Any community member who has no health professional designation or certification and who is not primarily employed in health-care delivery. This definition of laypeople excludes paraprofessional cadres such as community health workers, where emergency care formed part of the workers’ practice.

**Outcome criterion**

Individual or community health effects: Any quantified effect on morbidity, mortality or community capacity to manage a health problem. We considered willingness to provide emergency care as a health outcome when measured at the community or population level and not when measured only among trainees.

### Search strategy

We developed a search strategy to identify papers addressing first aid or prehospital emergency care by laypeople. We used a sample set of relevant articles to evaluate the recall and precision of search terms and refine our search strategy.^23^ Our search strategy is published elsewhere.^14^

We searched the following databases: MEDLINE®, Embase®, Cumulative Index to Nursing and Allied Health Literature (CINAHL), Scopus, SocINDEX, PsycINFO, Education Resources Information Center (ERIC), Cochrane Database of Systematic Reviews (CDSR), African Index Medicus (AIM), Index Medicus for the WHO Eastern Mediterranean Region (IMEMR), Latin American and Caribbean Health Sciences Literature (LILACS), Index Medicus for South-East Asia Region (IMSEAR) and Transport Research International Documentation (TRID). 

We adapted our search for grey literature using keywords that targeted the websites of humanitarian and global health agencies, academic grey literature databases, theses and dissertations, clinical trials registries and conference proceedings.^14^ We conducted our initial electronic search using the Google search engine on 17 March 2014 and searched all other sources on 3 May 2014. We later updated our search to include articles up to 16 December 2019. 

We also scanned the references of all included studies and manually searched references of first aid guidelines and reviews from the American Heart Association and the American Red Cross, European Resuscitation Council and International Liaison Committee on Resuscitation.^4^^,^^21^^,^^24^^–^^27^

### Study selection

We trained an international team of 18 reviewers with varied expertise in the subject matter and methods of the review, using a video to familiarize them with the research question and inclusion criteria. All reviewers screened a test set of 70 papers selected from our search that included seven papers that met enough inclusion criteria to proceed to full-text review. We conducted an internal study on this test set to confirm substantial interrater agreement (Fleiss’ *κ* >  0.61) between reviewers.^28^ More details are in the authors’ data respository.^17^ The reviewers screened the titles and abstracts of studies retrieved through the electronic and manual searches, independently and in duplicate. We conducted independent and duplicate full-text review of all papers retained through screening. One of the two lead investigators resolved discrepancies. We documented reasons when papers were excluded at this stage. We assessed papers in Dutch, English, French, German and Norwegian languages. We used Google Translate (Google LLC, Mountain View, United States of America, USA) and Cochrane TaskExchange volunteers^29^ to review papers in other languages.

### Data extraction

For each included paper, two investigators independently extracted information on the study objective, study design, population, details about the intervention and control groups (mode and duration of education; emergency health conditions treated; and role of the layperson), outcomes (type of health outcome; description of health outcome; type of emergency care provided; and effect size and confidence interval) and key conclusions. Where multiple publications reported on the same underlying study, we extracted data from all related papers and reported results from the most definitive paper.

We performed independent and duplicate assessment of study quality, including internal and external validity, selection and measurement biases, and confounding factors, using the Effective Public Health Practice Project quality assessment tool.^30^ This tool permits the appraisal of multiple types of studies and is designed and validated for the assessment of studies concerning health systems and population health interventions. We resolved discrepancies through consensus among the lead investigators.

### Synthesis

We prepared a narrative and tabular synthesis of our findings. We grouped studies qualitatively according to the illnesses or conditions addressed, the role of lay providers, the type of educational intervention provided and the type of outcomes reported. We distinguished individual health outcomes such as survival to hospital discharge; community health outcomes such as all-cause mortality; and measures of community capacity to manage emergencies such as cardiac arrest response times. Our rationale for these groupings was first to underscore the emergency health conditions for which studies had been identified, and then to provide information to guide future task shifting and first aid training interventions. We drew on Cochrane Collaboration guidance on syntheses without meta-analysis to assess the risk of bias across studies, and considered the number of studies, consistency of effects and directness of findings to develop plain-language summary statements of the effects of interventions.^31^

## Results

**Fig. 1:**
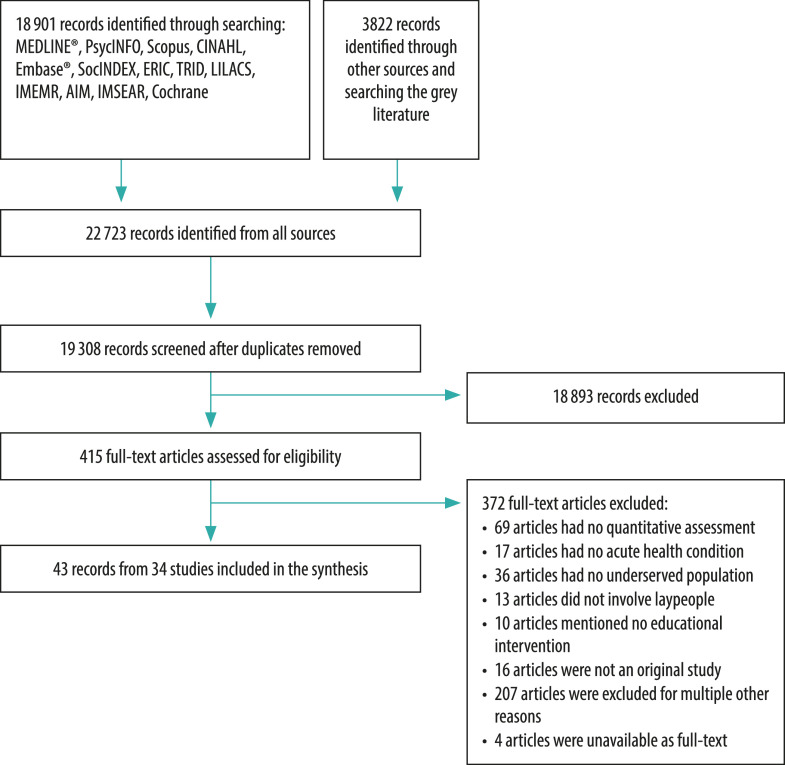
Flowchart of studies included in the systematic review of first aid by lay persons in low-resource settings and underserved populations

Our database searches yielded 19 308 unique papers. We retained 415 papers for full-text review, resulting in 43 eligible papers from 34 unique studies (Fig. 1). Grey literature and manual searches did not yield additional publications. Interrater agreement between the screening authors was good for study inclusion (Fleiss’ *κ* = 0.75).^17^ Studies excluded at full-text review are described in the authors’ data respository.^17^

### Study characteristics

Table 1 (available at: http://www.who.int/bulletin/volumes/99/7/20-270249) summarizes the studies that met the inclusion criteria, grouped by emergency medical condition, including cardiac arrest (four studies),^32^^–^^35^ burns (two studies),^36^^,^^37^ malaria (10 studies),^38^^–^^47^ severe malnutrition (one study),^48^ opioid poisoning (seven studies),^49^^–^^55^ paediatric communicable diseases (five studies),^56^^–^^60^ snakebites (one study),^61^ trauma (three studies)^62^^–^^64^ and various other emergencies (one study).^65^ The authors’ data repository provides more details of secondary outcomes and data from multiple reports arising from the same study.^17^

**Table 1:** Characteristics of studies included in the systematic review of health effects of first aid by lay responders in low-resource settings and underserved populations

Medical condition and study	Country	Study population and age	Study design and study period	Intervention	Control	Primary outcome and effect size	Quality rating^a^
**Cardiac arrest**							
Roberts et al., 1999^32^	United Kingdom	Population: approximately 30 000 people. Age: NR	Case series. Study period: 1 year	Intervention: training on basic life support for lay first responders. Participants: 83 people trained; 134 cardiac arrest patients treated	Control: none	Difference in mean response time to cardiac arrest calls between first responders and ambulances: 7.6 minutes^b^	Weak
Page et al., 2000^33^	USA and international airline flight routes	Population: 627 956 flights, 70 801 874 passengers. Age: mean 58 years, patients treated	Case series. Study period: 12.5 months	Intervention: appropriate use of automated external defibrillator on flights. Participants: 24 000 flight attendants trained; 200 patients treated, 15 of whom were defibrillated	Control: none	Percentage of patients alive at hospital discharge: 99/200 patients were unconscious; 40% (6/15 patients) survived neurologically intact to hospital discharge^b^	Weak
Rørtveit & Meland, 2010^34^	Norway	Population: 4400 people. Age: 36–92 years, patients treated	Case series. Study period: 5 years	Intervention: basic life support and defibrillation initiated by laypeople. Participants: 42 people trained; 17 patients treated among 24 cardiac arrest calls	Control: none	Median time from first responder arrival until ambulance or doctor arrival: 22.5 minutes^b^	Weak
Nielsen et al., 2013^35^^c^	Denmark	Population: 42 000 community members, 600 000 seasonal tourists annually. Age: > 15 years	Before-and-after study, uncontrolled. Study period: 1 year	Intervention: community-wide basic life support and automated external defibrillator use. Participants, number of people trained and treated: NR	Control: none	Percentage of community members willing to use an automated external defibrillator on a stranger: 63% (520/824 people) pre-intervention versus 82% (669/815 people) post-intervention (*χ^2^* test *P* < 0.0001; OR: 2.86; 95% CI: 2.26–3.63)^d^	Weak
Burns							
Sunder & Bharat, 1998^36^	India	Population: unknown. Age: 53.5% of inpatients age 25–35 years (frequencies not specified)	Before-and-after study, uncontrolled. Study period: 4 years	Intervention: occupational burn prevention and treatment education. Participants: 590 steel workers trained; 142 inpatients and 673 outpatients treated	Control: none	Percentage of burn patients with < 20% total body surface area burns receiving appropriate first aid: 37.8% (14/37 patients) pre-intervention versus 25.0% (4/16 patients) post-intervention; (OR: 3.75; 95% CI: 0.88–19.53)^d^	Weak
Skinner, et al., 2004^37^	New Zealand	Population: NR. Age: pre-intervention patients, 3 months to 77 years; post-intervention patients, 3 months to 83 years	Before-and-after study, uncontrolled. Study period: two 4-month study intervals, 44 months apart	Intervention: public first aid campaign for burn injuries. Participants: general public; number of people treated: NA	Control: none	Percentage of patients receiving adequate first aid: 33% (11/33^e^ people) pre-intervention versus 61% (22/36^e^ people) post-intervention (*P* = 0.02) among Pacific Islanders; 25% (6/24^e^ people) versus 48% (13/27^e^ people) post-intervention (*P* = 0.08) among Maori people	Moderate
**Malaria**							
Kidane & Morrow, 2000^38^	Ethiopia	Population: 37 regions, each with a population of 1000–3000 people; 14 001 children aged < 5 years. Age: < 5 years	Randomized controlled trial. Study period: 12 months	Intervention: peer education for mothers on recognition and treatment of paediatric malaria. Participants: 12 regions with 6383 children aged < 5 years; number of children treated: NR	Control: no peer education. Participants: 12 regions with 7294 children aged < 5 years; number of children treated: NR	Absolute rate reduction in all-cause mortality in children < 5 years: 20.4 per 1 000 (95% CI: 13.9–26.9)	Weak
Ajayi et al., 2008^39^	Nigeria	Population: 147 847 people, including 33 126 children and 33 576 women of childbearing age. Age: ≤ 10 years	Randomized controlled trial. Study period: 12 months	Intervention: peer education for mothers on paediatric malaria recognition and treatment. Participants: 330 mothers trained; 247 paediatric malaria cases treated	Control: no peer education. Participants: 281 mothers, 266 paediatric malaria cases	Percentage of children receiving chloroquine according to guideline on febrile illness for children at home: 2.6% (3/116 children) pre-intervention versus 52.3% (69/132 children) post-intervention (*P* < 0.001) in intervention group; 4.1% (3/72 children) pre-intervention versus 15.8% (9/57 children) post-intervention (*P* = 0.05) in control group	Weak
Kouyaté et al., 2008^40^	Burkina Faso	Population: NR. Age: < 5 years	Cluster randomized controlled trial. Study period: 2 years	Intervention: community-based malaria education and management. Participants: 70 women group leaders trained across 6 villages; 542 children treated at baseline and 496 children treated at follow-up	Control: no community-based malaria education and management. Participants: seven villages; 541 children treated at baseline and 510 children at follow-up	Percentage of children younger than 5 years with malaria with moderate to severe anaemia^f^: 28% (152 children) pre-intervention versus 17% (83 children) post-intervention in intervention group; 30% (162 children) versus 15% (74 children) post-intervention in control group (*P* = 0.32; OR: 1.18; 95% CI: 0.83–1.69)^d^	Weak
Ndiaye et al., 2013^41^	Senegal	Population: 40 000 people. Age: all ages	Case series. Study period: 4 years	Intervention: nurse-led education on malaria recognition and treatment. Participants: 31 community medicine distributors and 21 community health workers trained; 5384 consultations given by community medicine distributors and 16 757 by community health workers	Control: none	Percentage of eligible patients receiving rapid malaria tests: 93.5% (5036/5384 patients) treated by community medicine distributors; 56.8% (9518/16 757 patients) treated by community health workers^b^	Weak
Tobin-West & Briggs, 2015^42^	Nigeria	Population: 2187 people. Age: < 5 years	Before-and-after, controlled. Study period: 12 months	Intervention: community-based education on treatment of malaria. Participants: 184 mothers trained pre-intervention and 173 trained post-intervention; number treated: NR	Control: no training or drugs provided. Participants: 184 mothers pre-intervention and 169 post-intervention; number treated: NR	Percentage of mothers reporting their child was cured of malaria: 47.3% (87 mothers) pre-intervention versus 84.4% (146 mothers) post-intervention in intervention group (*P* < 0.0001); 50.0% (92 mothers) pre-intervention versus 49.1% (83 mothers) post-intervention in control group (*P* = 0.94)	Weak
Warsame et al., 2016^43^	Ghana, Guinea-Bissau, Uganda and United Republic of Tanzania	Population: 26 594 households, 346 villages; 58 771 children aged < 5 years; intervention: 141 clusters,12 297 households; control: 136 clusters, 10 531 households. Age: < 5 years	Cluster randomized controlled trial. Study period: 19 months	Intervention: community-based treatment for severe malaria before hospital referral. Participants: 687 mothers, traditional healers and others trained; 2464 children treated	Control: usual practice from community health workers. Participants: 1469 children treated	Odds ratio of initiation of malaria treatment in the community before hospital referral for severe malaria: 1.84 (95% CI: 1.20–2.83) trained mothers versus controls	Moderate
Kitutu et al., 2017^44^	Uganda	Population: 472 629 people; population aged < 5 years: NR. Age: < 5 years	Before-and-after, controlled. Study period: 12 months	Intervention: community-based treatment of various paediatric illnesses. Participants: owners and attendants at 61 drug shops trained; 212 caretaker–child pairs treated at baseline and 285 pairs treated at endline	Control: no community-based training. Participants: 23 drug shops; 216 caretaker–child pairs treated at baseline and 268 pairs treated at endline	Percentage of children younger than 5 years receiving guideline-based treatment for uncomplicated malaria: 8.3% (11/133 children) pre-intervention versus 57.5% (108/188 children) post-intervention in intervention group; 31.9% (38/119 children) pre-intervention versus 0.9% (1/112 children) post-intervention in control group. Difference between groups: 80.2% (95% CI: 53.2–107.2) of children received treatment	Weak
Linn et al., 2018^45^	Myanmar	Population: 978 735 people. Age: < 5 years (9.5%); 5–14 years (18.6%); ≥ 15 years (72.0%)	Cohort study, retrospective. Study period: 1 year	Intervention: screening, testing and management of malaria by village health volunteers, with referrals as needed. Participants: 270 155 volunteers trained; 23 503 (80.9%) patients received complete treatment	Control: similar to intervention, but conducted by basic health staff. Participants: 708 580 volunteers trained; 64 879 patients (88.2%) received complete treatment	Adjusted prevalence ratio of receiving malaria treatment among eligible patients in intervention versus control: 1.02 (95% CI: 1.015–1.020)	Weak
Green et al., 2019^46^	Zambia	Population: intervention area of 54 000 people in Serenje district. Age: < 5 years	Before-and-after study, uncontrolled. Study period: 12 months	Intervention: treatment and transport of children with severe paediatric malaria by community volunteers. Participants: 180 Safe Motherhood Action Group volunteers and 45 volunteers trained in integrated community case management, and 66 bicycle ambulance riders trained in emergency transport; 224 children treated before intervention and 619 children during intervention	Control: none	Malaria case fatality rate in children younger than 5 years: 8% (18/224 children) before intervention: 0.5% (3/619 children) during intervention^b^	Weak
Minn et al., 2019^47^	Myanmar	Population: 257 700 people. Age: all ages	Cross-sectional. Study period: 1 year	Intervention: malaria screening, diagnosis and treatment services by integrated community malaria volunteers, with referrals as appropriate. Participants: 632 volunteers trained; 2279/2881 (79%) of malaria-positive patients treated	Control: care from basic health staff at health posts	Adjusted probability ratio of receiving incorrect treatment for malaria from volunteers versus care at health posts: 0.5 (95% CI: 0.30–0.83)	Weak
**Malnutrition**							
Alé et al., 2016^48^	Niger	Population: intervention group, 37 389 people and 9908 children aged < 5 years; control group, 33 449 people and 8867 children aged < 5 years. Age: < 5 years	Non-randomized cluster trial. Study period: 11 months	Intervention: training on screening for severe acute malnutrition by mothers and caretakers. Participants: 12 893 mothers and caretakers trained; 1371 children admitted to malnutrition treatment	Control: screening for severe acute malnutrition by community health workers. Participants: 36 community health workers trained; 988 children admitted to malnutrition treatment	Percentage of children hospitalized for malnutrition treatment: 7.2% (99/1371 children) in intervention group versus 11.8% (117/988 children) in control group. Relative risk ratio of hospitalization: 0.61 (95% CI: 0.47–0.79); risk difference: −4.62% (95% CI: −7.06 to −2.18)	Weak
**Opioid poisoning**							
Walley et al., 2013^49^	USA	Population: 30% of population of Massachusetts State. Age: NR	Time-series analysis. Study period: 8 years, 2002–2009	Intervention: overdose education and naloxone distribution. Participants: 2912 people enrolled in training; 327 rescue attempts made	Control: none	Adjusted rate ratio relative to reference population with 0 enrolments per 100 000 population: 0.73 (95% CI: 0.57–0.91) in regions with 1–100 enrolments in training per 100 000 population; 0.54 (95% CI: 0.39–0.76) in regions with > 100 enrolments in training per 100 000 population	Weak
Bird et al., 2016^50^	Scotland, United Kingdom	Population: about 5.1 million people; affected sub-population size: NR. Age: NR	Before-and-after study, uncontrolled. Study period: 2006–2010 pre-intervention, 2011–2013 post-intervention	Intervention: nationwide education on opioid overdose and naloxone distribution programme. Participants: 11 898 kits issued by community and prisons; numbers of patients treated unknown	Control: none	Percentage of opioid-related deaths with a 4-week antecedent of prison release: 9.8% (193/1970 people) pre-intervention versus 6.3% (76/1212 people) post-intervention (absolute difference: 3.5%; 95% CI: 1.6–5.4%)	Moderate
Irvine et al., 2019^51^	British Columbia, Canada	Population: not specified (population of British Columbia). Age: NR	Cohort study, retrospective, with Markov chain modelling. Study period: about 20 months (Apr 2016–Dec 2017)	Intervention: provincial distribution of naloxone kits, as well as provincial overdose prevention and supervised consumption services and opioid agonist therapy. Participants: 88 300 naloxone kits distributed in 2017; number of patients treated unknown	Control: none	Number of opioid-related-deaths averted:1650 (95% CrI: 1540–1850); 11 kits used per death averted (95% CrI: 10–13)	Moderate
Mahonski et al., 2020^52^	Maryland, USA	Population: 1139 people with opioid poisoning and community naloxone administration. Age: all ages, mean age 34.3 years	Cohort study, retrospective. Study period: 24 months, Jan 2015–Oct 2017	Intervention: overdose education and naloxone distribution. Participants: 70 992 people trained in 2015–2017, including 6031 law enforcement officers; 1139 patients treated	Control: none	Percentage of opioid poisoning cases reversed: 79.2% of 886 poisoning cases overall; decrease from 82.1% (96/117 patients) in 2015 to 76.4% (441/577 patients) in 2017 (*P* = 0.04)	Weak
Naumann et al., 2019^53^	North Carolina, USA	Population: not specified (population of North Carolina State). Age: NR	Before-and-after, uncontrolled. Study period: 2000–2016	Intervention: overdose education and naloxone distribution. Participants: 39 449 naloxone kits distributed; numbers treated unknown	Control: none	Rate ratio of opioid poisoning deaths in intervention counties compared with counties not receiving naloxone kits: 0.90 (95% CI: 0.78–1.04) in counties with 1–100 kits distributed per 100 000 population; 0.88 (95% CI: 0.7–1.02) in counties with > 100 kits distributed per 100 000 population	Weak
Papp et al., 2019^54^	North-east Ohio, USA	Population: 291 people who use opioids. Age: median 34 years	Cohort study, retrospective. Study period: 3 and 6 months from hospital discharge	Intervention: hospital-based overdose education and naloxone distribution. Participants: 208 (71%) overdose survivors trained; treatment outcome reported among trainees	Control: no overdose education or naloxone distribution. Participants: 83 overdose survivors untrained; number of patients treated: NA	Percentage of patients experiencing repeat overdose-related emergency department visit, hospitalization or death (composite of events): 6.0% (5/83 patients) in control group versus 7.7% (16/208 patients) in intervention group over 3 months (*P* = 0.9); 4.8% (4/83 patients) in control group versus 6.7% (14/208 patients) in intervention group over 6 months (*P* = 0.99)	Weak
Rowe et al., 2019^55^	San Francisco, USA	Population: not specified (population of San Francisco). Age: NR	Before-and-after, uncontrolled. Study period: 2014–2015	Intervention: overdose education and naloxone distribution. Participants: 1023 overdose education and naloxone distribution trainees in 2014 and 1123 trainees in 2015; 326 people trained in 2014 and 504 trained in 2015	Control: none	Number of opioid poisoning reversals reported: 326 in 2014 versus 504 in 2015 (*P* < 0.001)	Weak
**Paediatric communicable diseases**							
Bang et al., 1994^56^	India	Population: 48 377 people in 58 villages in intervention area; 34 856 people in 44 villages in control area. Age: < 5 years	Non-randomized cluster trial. Study period: 3 years	Intervention: management of childhood pneumonia by lay community members. Participants: 30 paramedical workers, 25 village health workers and 86 traditional birth attendants trained (only traditional birth attendants met layperson inclusion criterion); traditional birth attendants managed 651 cases of pneumonia among children aged < 5 years and 50 cases among neonates	Control: existing care. Participants: no community members trained; number of children treated unknown	Pneumonia case fatality rate in children younger than 5 years: 2.0% (13/651 children) with care by traditional birth attendants versus 13.5% with existing care (frequencies: NR)	Moderate
Holloway et al., 2009^57^	Nepal	Population: 4 districts of 134 000–232 000 people each; population aged < 5 years unknown. Sample frame of 2231 households with a child aged < 5 years old who had acute respiratory infection in last 2 weeks. Age: < 5 years	Before-and-after, controlled. Study period: about 6 months	Intervention: community-wide education programme on recognizing and treating acute respiratory infections. Participants: community exposed to public campaign; 200 children aged < 5 years with severe acute respiratory infection treated	Control: existing care. Participants: community not exposed to campaign; 187 children aged < 5 years with severe acute respiratory infection treated	Absolute difference in percentage of children younger than 5 years with severe acute respiratory infection receiving consultation at a health post: 12.6 % (test of interaction with intervention versus control group *P* = 0.01)	Weak
Yansaneh et al., 2014^58^	Sierra Leone	Population: projected 57 000–76 000 children (19% of 300 000–400 000 people). Age: < 5 years	Before-and-after, controlled. Study period: 2 years	Intervention: treatment and referral of common childhood illnesses by lay volunteers. Participants: 2129 volunteers trained; 1980 children brought for medical care at baseline and 1657 patients at endline	Control: existing care. Participants: no people trained; 1962 patients brought for care at baseline and 2102 patients at endline	Odds ratio of appropriate treatment: 0.45 (95% CI: 0.21–0.96) for childhood diarrhoea; 0.65 (95% CI: 0.32–1.34) for malaria; 2.05 (95% CI: 1.22–3.42) for pneumonia	Weak
Langston et al., 2019^59^	Province of Tanganyika, Democratic Republic of the Congo	Population: 2 649 317 people. Age: NR	Non-randomized cluster trial. Study period: 11 months	Intervention: simplified teaching of integrated community case management for uncomplicated malaria, pneumonia and diarrhoea for children aged 2–59 months. Participants: 1600 people trained and 78 lay providers assessed; 78 children assessed	Control: standard teaching for integrated community case management of uncomplicated malaria, pneumonia and diarrhoea. Participants: 74 lay providers assessed; 74 children assessed	Adjusted odds ratio of correct referral of children with danger signs: 24.2 (95% CI: 1.9–300.2)	Moderate
Oresanya et al., 2019^60^	Niger State, Nigeria	Population: 899 sick children from caregiver survey included at baseline and 680 sick children at endline. Age: < 5 years	Before-and-after, uncontrolled. Study period: from baseline 2014 to endline 2017	Intervention: treatment and management of paediatric diarrhoea, pneumonia and fever by volunteer community caregivers. Participants: 1320 volunteers trained; 161 patients treated	Control: none	Percentage of children younger than 5 years brought for care to an appropriate provider: for fever, 78% (322/413 children) at baseline versus 94% (283/301 children) at endline, (*P* < 0.01); for diarrhoea, 72% (269/374 children) at baseline versus 91% (274/300 children) at endline (*P* < 0.01);for pneumonia, 76% (262/343 children) at baseline versus 89% (267/301 children) at endline (*P* < 0.05)	Moderate
**Snakebites**							
Sharma et al., 2013^61^	Nepal	Population: 60 759 people pre-intervention; 59 383 people post-intervention. Age: NR	Before-and-after study, uncontrolled. Study period: Nov–Dec 2003 versus Nov–Dec 2004	Intervention: community-wide campaign to promote snakebite awareness and rapid transport. Participants: 10 motorcycle drivers trained in each of four subregions; two to three public snakebite awareness programmes per subregion, numbers attending unspecified; leaflets, banners and posters distributed; 122/305 snakebite patients transported by motorcycle pre-intervention, 143/187 during intervention	Control: none	Snakebite case fatality rate: 10.5% (32/305 people) pre-intervention versus 0.51% (187 people) post-intervention; relative risk reduction: 0.95 (95% CI: 0.70–0.99); absolute risk reduction: 10.04 (95% CI: 7.38–15.72)^e^	Weak
**Trauma**							
Husum et al., 2003^62^^c^	Cambodia and Iraq	Population: NR. Age: NR	Before-and-after study, uncontrolled. Study period: 5 years from 1997 to 2001	Intervention: trauma first aid administered by lay responders. Participants: 135 paramedics and 5237 lay responders trained; 224/1285 emergency medical patients and 1061/1285 trauma patients treated	Control: none	Absolute change in physiological severity score from prehospital to hospital arrival: 0.3 at baseline versus 0.7 after intervention; difference in differences: 0.4 (95% CI: 0.2–0.6).	Strong
Saghafinia et al., 2009^63^^c^	Iran (Islamic Republic of)	Population: not specified. Age: mean 31.9 years	Cohort study, prospective. Study period: 4 years	Intervention: pre-hospital first aid provided by lay individuals. Participants: 4834 lay villagers, nomads and various clinicians trained; 152/288 patients received prehospital care; 63/288 patients died before reaching hospital	Control: no prehospital treatment of injured people; patients moved directly to the hospital. Setting same as intervention group. Participants: no people trained; 73/288 patients sent directly to hospital.	Mean physiological severity scores: 6.40 prehospital versus 7.43 at hospital arrival (95% CI: −0.72 to −0.45) in intervention group; 5.97 in control group	Weak
Murad et al., 2012^64^^c^	Iraq	Population: NR. Age: mean 26 years in survivors, 27 years in non-survivors	Before-and-after study, uncontrolled. Study period: 10 years	Intervention: prehospital trauma care delivered by lay responders. Participants: 7000 layperson first helpers trained; 2788 patients treated	Control: none	Mortality among trauma patients receiving treatment: 17% (95% CI: 15–19) pre-intervention versus 4% (95% CI: 3.5–5) post-intervention (frequencies: NR)	Moderate
Various emergencies							
Lavallée et al., 1990^65^	Canada	Population: about 3000 people. Age: NR	Before-and-after study, controlled. Study period: 1 year	Intervention: distribution of medical kits and first aid training to Indigenous hunters in wilderness camps. Participants: 210 volunteers trained (49% participation rate across communities); number of people treated unknown	Control: no medical kits and first aid training. Setting same as intervention group. Participants: number of people trained NA; number of people treated: NA	Percentage of emergency health cases managed at wilderness hunt camps with kit: 60% versus 36% without kit^b^ (frequencies: NR)	Weak

CI: confidence interval; CrI: credible interval; NA: not applicable; NR: not reported; OR: odds ratio.

^a^ We used the Effective Public Health Practice Project quality tool to assess internal and external validity, selection and measurement biases, and confounding factors.^30^

^b^ Test of significance was not reported and we could not compute significance appropriately from the reported data.

^c^ We retrieved multiple papers regarding the same study. See the authors' data respository.^17^

^d^ We computed Fisher exact test using the reported data.

^e^ We computed values based on the reported data.

^f^ Haematocrit ≤ 24%.

Most studies used observational or quasi-experimental designs, including 11 uncontrolled before-and-after studies,^35^^–^^37^^,^^46^^,^^50^^,^^53^^,^^55^^,^^60^^–^^62^^,^^64^ five controlled before-and-after studies,^42^^,^^44^^,^^57^^,^^58^^,^^65^ one prospective cohort study,^63^ four retrospective cohort studies,^45^^,^^51^^,^^52^^,^^54^ four case series,^32^^–^^34^^,^^41^ three non-randomized cluster trials,^48^^,^^56^^,^^59^ one interrupted time-series analysis^49^ and one cross-sectional study.^47^ Experimental studies included two randomized controlled trials^38^^,^^39^ and two cluster randomized controlled trials.^40^^,^^43^

**Fig. 2:**
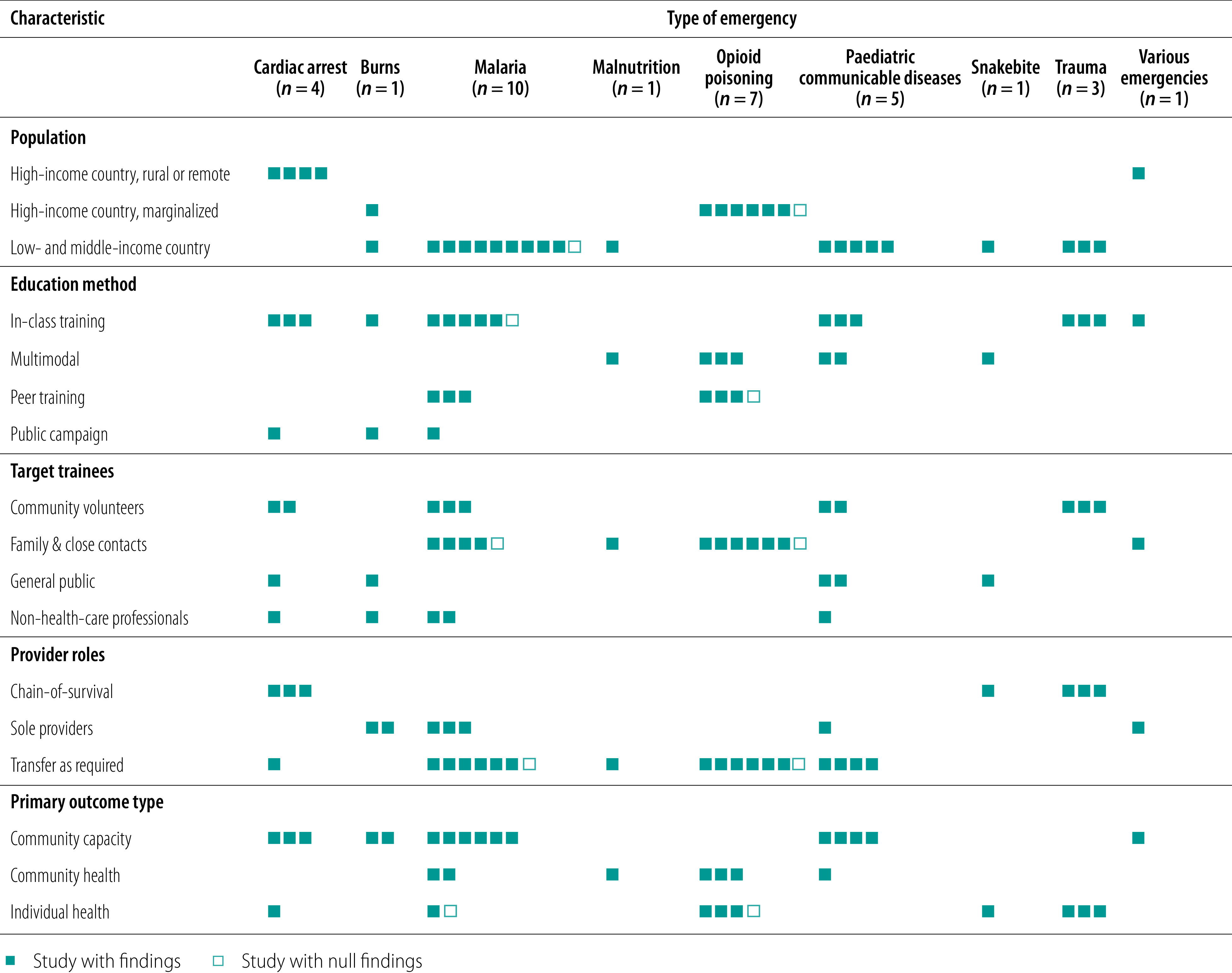
Summary characteristic and training interventions for first aid by lay responders in low-resource settings and underserved populations

Note: Each individual study is represented by a block.

The populations studied included rural, urban and underserved subpopulations from North America, Europe, Asia, Africa and Australia and Oceania. Sample sizes ranged from under 300 people to population-based studies of over 5 million people (Table 1). Table 2 and Fig. 2 provide a description of the training interventions, demonstrating the diversity of populations, interventions, target trainees, provider roles and primary outcome types for each study across each emergency health condition.^66^ Twenty-one studies (62%) were conducted in low- and middle-income countries, including all studies concerning interventions for malaria, paediatric communicable diseases, malnutrition and trauma.^36^^,^^38^^–^^48^^,^^56^^–^^64^ Studies conducted in high-income countries studied underserved rural populations and marginalized communities such as people who use drugs or Indigenous peoples.^32^^–^^35^^, ^^37^^,^^49^^–^^55^^,^^65^ With the exception of burns, which was studied in both a middle-income country (India) and an underserved population in a high-income country (New Zealand Maori and Pacific Islanders), interventions were studied in either low- and middle-income countries or high-income countries, but not both.^36^^,^^37^ For example, all studies concerning cardiac arrest were in high-income countries, while all studies concerning physical trauma were in low- and middle-income countries.

**Table 2:** Summary of training interventions for first aid by laypeople in low-resource settings and underserved populations

Medical condition and study	Study setting^a^	Education modality	Target trainees	Provider roles	Primary outcome type	Training description (study design)
**Cardiac arrest**						
Roberts et al., 1999^32^	Rural or remote population in high-income country	In-class training	Community volunteers	Chain-of-survival	Community capacity	8-hour cardiopulmonary resuscitation and first aid course. (Case series, no control)
Page et al., 2000^33^	Rural or remote population in high-income country	In-class training	Non-health-care professionals	Transfer as required	Individual health	4-hour cardiopulmonary resuscitation and automated external defibrillator workshop, and 1.5-hour refresher for commercial aircraft flight attendants. (Case series, no control)
Rørtveit & Meland, 2010^34^	Rural or remote population in high-income country	In-class training	Community volunteers	Chain-of-survival	Community capacity	Basic life support and automated external defibrillator course; course duration; NR. (Case series, no control)
Nielsen et al., 2013^35^	Rural or remote population in high-income country	Public campaign	General public	Chain-of-survival	Community capacity	24-minutes long video-based basic life support self-training kits offered year-long; 4-hour basic life support and automated external defibrillator course; local news broadcasted cardiac arrest information and course offerings. (No separate control training)
**Burns**						
Sunder & Bharat, 1998^36^	Low- or middle-income country	Public campaign	Non-health-care professionals	Sole providers	Community capacity	Annual 75-minute audio-visual session for industrial steel workers on burns safety and first aid; 6 sessions per year. (No separate control training)
Skinner et al., 2004^37^	Marginalized community in high-income country	In-class training	General public	Sole providers	Community capacity	Multimedia advertisements including television, radio, billboards, newspapers and magazines on burn injuries and first aid; campaign duration: NR. (No separate control training)
**Malaria**						
Kidane & Morrow, 2000^38^	Low- or middle-income country	Peer training	Family and close contacts	Transfer as required	Community health	Mothers taught to recognize malaria, to administer chloroquine and recognize adverse reactions; referrals through mother trainers; training duration: NR. (No peer education)
Ajayi et al., 2008^39^	Low- or middle-income country	Peer training	Family and close contacts	Transfer as required	Community capacity	Mothers trained on malaria treatment; pictorial guideline distributed; training duration: NR. (No peer education)
Kouyaté et al., 2008^40^^,b^	Low- or middle-income country	In-class training	Family and close contacts	Transfer as required	Individual health	5-day training course and 1-day refresher for mothers; discussions and role-play on malaria management and chloroquine administration. (No community-based malaria education and management)
Ndiaye et al., 2013^41^	Low- or middle-income country	In-class training	Non-health-care professionals	Sole providers	Community capacity	3-day classroom teaching and 15-day training at health post on malaria identification, use of rapid malaria tests, artemisinin-based combination therapy, and to recognize adverse reactions. (Case series, no control)
Tobin-West & Briggs, 2015^42^	Low- or middle-income country	Peer training	Family and close contacts	Sole providers	Individual health	12 hours of training over 4 days for mothers, covering malaria prevention, recognition and management. (No training or drugs provided)
Warsame et al., 2016^43^	Low- or middle-income country	Public campaign	Family and close contacts	Transfer as required	Community capacity	Community posters on recognition of severe malaria, suppository administration and referral; campaign duration: NR. (Usual practice by community health workers)
Kitutu et al., 2017^44^	Low- or middle-income country	In-class training	Non-health-care professionals	Sole providers	Community capacity	Drug sellers trained to test for and treat uncomplicated malaria, pneumonia symptoms and non-bloody diarrhoea; training duration: NR. (No community-based training)
Linn et al., 2018^45^	Low- or middle-income country	In-class training	Community volunteers	Transfer as required	Community capacity	5-day modular training on screening, testing and management of malaria, including referrals provided to village health volunteers. (No separate control training)
Green et al., 2019^46^	Low- or middle-income country	In-class training	Community volunteers	Transfer as required	Community health	Volunteers trained to administer rectal artesunate to children showing signs of severe malaria and refer appropriately, and train-the-trainer cascade model; training duration: NR. (No separate control training)
Minn et al., 2019^47^	Low- or middle-income country	In-class training	Community volunteers	Transfer as required	Community capacity	9-day training on the danger signs, diagnosis, treatment and recording/reporting of malaria, as well as the signs and symptoms of tuberculosis; health education on dengue, filariasis, sexually transmitted infection, HIV and leprosy; with annual refresher training. (No separate control training)
**Malnutrition**						
Ale et al., 2016^48^	Low- or middle-income country	Multimodal	Family and close contacts	Transfer as required	Community health	Group sessions of <1 day with up to 30 mothers or caretakers; brief home-based training on consent and screening for malnutrition. (Community health workers received theory and practical training on malnutrition screening, awareness, and referral)
**Opioid poisoning**						
Walley et al., 2013^49^	Marginalized community in high-income country	Multimodal	Family and close contacts	Transfer as required	Community health	10–60 minutes of overdose education and naloxone distribution training conducted in groups or individually, focusing on overdose prevention and naloxone administration for people who use opioids or are likely to witness overdose. (No separate control training)
Bird et al., 2016^50^	Marginalized community in high-income country	Multimodal	Family and close contacts	Transfer as required	Individual health	10–15 minutes of in-person, face-to-face education on intramuscular administration of naloxone and overdose first aid for people who use opioids or are likely to witness overdose. (No separate control training)
Irvine et al., 2019^51^	Marginalized community in high-income country	Multimodal	Family and close contacts	Transfer as required	Individual health	British Columbia's take-home naloxone kit programme for people who use opioids or are likely to witness an overdose; training duration: NR. (No separate control training)
Mahonski et al., 2020^52^	Marginalized community in high-income country	Multimodal	Family and close contacts	Transfer as required	Individual health	State-sponsored education on overdose recognition, contacting emergency medical services and how to assemble and administer an intranasal naloxone device; training duration: NR. (No separate control training)
Naumann et al., 2019^53^	Marginalized community in high-income country	Peer training	Family and close contacts	Transfer as required	Community health	Community-based education on overdose and naloxone administration, education on Good Samaritan law; training duration: NR. (No separate control training)
Papp et al., 2019^54^^,b^	Marginalized community in high-income country	Peer training	Family and close contacts	Transfer as required	Individual health	One-on-one hospital-based overdose education and naloxone distribution for people treated for heroin overdose in the emergency department; training duration: NR. (No overdose education and naloxone distribution)
Rowe et al., 2019^55^	Marginalized community in high-income country	Peer training	Family and close contacts	Transfer as required	Community health	Community-based education on identifying and managing an opioid overdose and intramuscular or intranasal naloxone administration; training duration: NR. (No separate control training)
**Paediatric communicable diseases**						
Bang et al., 1994^56^	Low- or middle-income country	In-class training	Non-health-care professionals	Transfer as required	Community health	Six classes of 1.5 hours each to train traditional birth attendants who did not traditionally provide baby care to recognize childhood pneumonia, administer pharmacotherapy, and refer as needed. (No separate control training)
Holloway et al., 2009^57^	Low- or middle-income country	Multimodal	General public	Sole providers	Community capacity	3-day training for teachers and district health staff; 10-day workshop for students and other community members. Community posters and street theatre about acute respiratory infections. (No separate control training)
Yansaneh et al., 2014^58^	Low- or middle-income country	In-class training	Community volunteers	Transfer as required	Community capacity	1-week training on symptomatic malaria, pneumonia and diarrhoea and appropriate treatment for each. Also trained to recognize severe symptoms and refer to health centres. (No separate control training)
Langston et al., 2019^59^	Low- or middle-income country	In-class training	Community volunteers	Transfer as required	Community capacity	6-day training on simplified version of curriculum (four data collection tools) for various paediatric illnesses; focused on practical training through role-play and discussions. (Similar to intervention, but standard version of curriculum which includes seven data collection tools)
Oresanya et al., 2019^60^	Low- or middle-income country	Multimodal	General public	Transfer as required	Community capacity	Community volunteers trained to recognize, treat, document and refer children as needed; community mobilization efforts including mass media campaigns, and community dialogues were also undertaken to promote care-seeking, uptake of services, and promote services offered by community volunteers. (No separate control training)
**Snakebites**						
Sharma et al., 2013^61^	Low- or middle-income country	Multimodal	General public	Chain-of-survival	Individual health	Snakebite awareness sessions, leaflets, banners and posters. Emphasis on rapid transport of victims to the nearest treatment centre. <1 day of training for motorcycle drivers; two to three snakebite awareness sessions for other community members. (No separate control training)
**Trauma**						
Husum et al., 2003^62^	Low- or middle-income country	In-class training	Community volunteers	Chain-of-survival	Individual health	2-day course on basic first aid for village first responders; 1-day rehearsal training after 6–12 months. (No separate control training)
Saghafinia et al., 2009^63^	Low- or middle-income country	In-class training	Community volunteers	Chain-of-survival	Individual health	15-hour basic trauma care courses for people with higher education and teachers; 12-hour first aid courses for people with lower education and high school students; and 8-hour brief courses for laypersons and refresher courses every month. (No separate control training)
Murad et al., 2012^64^	Low- or middle-income country	In-class training	Community volunteers	Chain-of-survival	Individual health	2-day instructional class for lay responders on basic trauma care. (Paramedics were trained to provide trauma life support in the field and during evacuations, and were also trained to teach basic life support to laypersons)
**Various emergencies**						
Lavallée et al., 1990^65^	Rural or remote population in high-income country	In-class training	Family and close contacts	Sole providers	Community capacity	30-hour training course and manual in bush kits for hunters and trappers. (No training and bush kits provided)

HIV: human immunodeficiency virus; NR: not reported.

^a^ Income groups are World Bank classifications.

^b^ Studies with null findings.

### Study interventions

The included studies described a variety of educational approaches, including public campaigns (three studies), in-class training programmes (17 studies), peer or individual training (seven studies) and multimodal training programmes (seven studies). A total of 33 studies assessed targeted interventions addressing priority emergency health conditions in the given population, such as opioid poisoning among people who use drugs or trauma management in regions with an elevated incidence of trauma from landmines. One study assessed a comprehensive training initiative designed to enhance responses to diverse conditions among Indigenous hunters and trappers in remote Canada (Fig. 2).^65^

Trainees included the general public (five studies), non-health-care professionals such as drug retailers and flight attendants (five studies), community volunteers (10 studies) and family members and close contacts of at-risk populations such as people who use opioids or children at risk of malaria (14 studies; Fig. 2). We identified five studies evaluating the impact of training mothers to respond to emergency health conditions in children, including malaria and malnutrition.^38^^,^^39^^,^^42^^,^^43^^,^^48^

Trainees were taught to attend to emergencies as sole providers (seven studies), as sole providers with responsibility for transferring selected patients to other professionals (20 studies), or as responders in a community chain-of-survival involving routine patient transfer to other providers (seven studies; Fig. 2).

Studies reported most commonly on measures of community capacity to manage health emergencies (14 studies), while 13 studies reported on individual health and seven reported on community health outcomes (Fig. 2).

### Outcomes

Most studies reported small effect sizes (Table 1). Some studies reported statistically significant and clinically important effects on measures of individual and community health. For example, one study reported an absolute reduction of 20.4 per 1000 in all-cause under-5 mortality in a randomized trial of malaria peer education for mothers in Ethiopia.^38^ Another study comprising 2788 patients treated for trauma reported a reduction in mortality from 17% to 4% in a before-and-after study of a community first-responder programme in Iraq.^64^ Through a cohort and modelling study, researchers estimated that opioid overdose education and naloxone distribution in British Columbia, Canada, averted 1650 deaths in 20 months.^51^

Two included papers reported null or equivocal results. A cluster randomized controlled trial of a malaria education and management programme for women’s groups observed no effect on the prevalence of severe malaria-associated anaemia in children.^40^ A cohort study of overdose education and naloxone distribution among emergency department patients in Ohio, USA, found no statistically significant reduction in the composite outcome of overdose-related emergency department visits, hospitalizations or deaths.^54^

Table 3 summarizes our findings across studies for each health condition and provides a global synthesis across all included conditions, with the risk of bias across studies for each summary statement. Studies were predominantly of weak (24 studies) or moderate (nine studies) quality. We included one study with methods rated as strong quality concerning prehospital trauma care (Table 1). The authors’ data repository provides detailed component quality ratings.^17^^,^^30^

**Table 3:** Summary of findings of the systematic review of first aid by lay responders in low-resource settings and underserved populations

Medical condition, outcome type and outcome	No. of studies per outcome	Impact	Overall quality^a^
**Cardiac arrest**			
Community capacity			
Willingness to use an automated external defibrillator	1	Community-wide training on basic life support or automated external defibrillator use in rural and remote settings may improve public willingness to provide some aspects of cardiopulmonary resuscitation and automated external defibrillator use^35^	Weak
First response time	2	Lay responders with training on basic life support may provide faster cardiac arrest response times than professional responders in rural settings^32^^,^^34^	Weak
Individual health			
Survival at hospital discharge	1	Training on automated external defibrillator use by flight attendants may improve cardiac arrest survival on commercial aircraft^33^	Weak
**Burns**			
Community capacity			
Appropriate initial first aid	2	Burns education campaigns may improve appropriate first aid for burns in underserved populations and people at elevated occupational risk of burns^36^^,^^37^	Weak
**Malaria**			
Community health			
Under-5 all-cause mortality	1	Peer and volunteer education on paediatric malaria recognition and treatment may reduce all-cause under-5 mortality and case-fatality rates in rural low-income malaria-endemic settings^38^^,^^46^	Weak
Under-5 malaria case fatality rate	1		
Community capacity			
Appropriate diagnosis and treatment of paediatric malaria	6	Training laypeople such as mothers, community volunteers and lay drug vendors to identify and treat acute paediatric malaria may improve local capacity to diagnose and treat malaria appropriately in low-income settings^39^^,^^41^^,^^43^^–^^45^^,^^47^	Weak
Individual health			
Proportion of moderate to severe anaemia in children under 5 years old (null findings^40^)	1	The evidence does not refute and may support the effectiveness of community-based acute malaria education and management programmes to improve malaria severity and cure rates in low-income malaria-endemic settings^40^^,^^42^	Weak
Number of patients cured of malaria	1		
**Malnutrition**			
Community health			
Hospitalization	1	Training mothers and caretakers to screen for severe paediatric malnutrition in low-income settings may reduce hospitalization rates for severe malnutrition^48^	Weak
**Opioid poisoning**			
Community health			
Overdose-related deaths	2	Naloxone distribution programmes may result in lower rates of opioid-overdose deaths and more opioid poisoning reversals than communities with less naloxone distribution uptake^49^^,^^53^^,^^55^	Weak
Opioid poisoning reversals	1		
Individual health			
Overdose deaths	2	Naloxone distribution programmes may result in the prehospital reversal of opioid poisonings and avert opioid-related deaths^50^^–^^52^^,^^54^	Weak
Composite of repeat overdose-related emergency department visit, hospitalization, or death (null findings)^54^	1		
% of opioid poisoning cases reversed	1		
**Paediatric communicable diseases**			
Community health			
Pneumonia-specific fatality rate	1	Community-wide education and management of paediatric acute respiratory infections may reduce pneumonia-specific fatality rates and improve access to treatment services in rural settings^56^	Weak
Community capacity			
Appropriate consultation and referral to health-care services	3	Community-wide education and management of paediatric acute respiratory infections may improve access to treatment services in rural settings^57^^–^^60^	Weak
Appropriate treatment by symptom	1		
**Snakebites**			
Individual health			
Bite-specific mortality	1	Community snakebite education campaigns in low-resource settings with a high burden of snakebite fatalities may reduce snakebite case fatality rates^61^	Weak
**Trauma**			
Individual health			
Trauma-specific mortality	1	Trauma first aid training for lay responders slightly improves physiological severity scores on presentation to hospital and is likely to reduce trauma mortality in remote and low-resource settings with elevated injury rates^62^^–^^64^	Moderate
Physiological severity score on presentation to hospital	2		
**Various emergencies**			
Community capacity			
Percentage of patients managed in remote settings	1	Medical training and kits for Indigenous hunters and trappers may improve field management of common health problems and reduce air evacuations from remote hunting and trapping camps^65^	Weak
**Global synthesis**			
All			
Various	34	First aid education and task shifting to laypeople may improve patient morbidity and mortality and community capacity to manage health emergencies for some adult and paediatric acute conditions, including cardiac arrest, burns, malaria, malnutrition, opioid poisoning, paediatric communicable diseases, snakebites and trauma	Weak

^a^ Where there were multiple studies, we examined the ratings across studies, weighing the evidence of different studies, and then downgraded the quality score of studies as required before deciding on the total risk of bias.

## Discussion

We found that first aid education and task shifting to laypeople may reduce morbidity and mortality, and enhance community capacity to manage health emergencies for a variety of emergency conditions. The studies include cardiac arrest, burns, malaria, malnutrition, opioid poisoning, paediatric communicable diseases, snakebites and trauma. All of the included studies evaluated targeted training for priority local emergency conditions; there were no eligible studies concerning courses with general, untargeted first aid curricula. The overall weak quality of studies in our review underscores the limitations in the available science, the need for rigorous studies in this field, and the challenges inherent in evaluating complex population health interventions such as task shifting.^67^ The widespread practice of training laypeople to deliver lifesaving interventions for acute health emergencies in underserved settings arises from sound logic and humanitarian principles.^2^^,^^6^^,^^7^ Our review shows that there is limited empirical evidence to demonstrate an individual or community health benefit arising from this practice. 

Previous reviews demonstrate the effectiveness of first aid education by reporting on knowledge, skills, helping behaviours or confidence among trainees.^8^ Guidelines and curricula for first aid generally derive interventions for the lay public by adapting practices from professional prehospital practice and health-care research.^21^^,^^26^ Database searches that rely on the keywords “first aid” or “layperson” to retrieve studies concerning first aid may overlook papers concerning interventions that do not use these terms but are thematically aligned with first aid. In comparison with other reviews on first aid, ours covers a greater breadth of research concerning interventions provided by laypeople to address emergency medical problems in underserved populations and low-resource settings.^68^^,^^69^

Like other task-shifting strategies, first aid education is a complex, system-level intervention that requires its own foundation of evidence.^70^ Clinical interventions that may be effective when implemented by professionals may not produce the same results when implemented by other providers. The adaptation of professional practices and the assessment of educational outcomes among people trained in first aid is insufficient to establish the effectiveness and safety of first aid interventions for target patients, programmes or communities.

Our review advances novel conceptual ties between first aid and task shifting. Lay emergency care and volunteer paramedic interventions are among the most cost-effective ways to reduce avoidable mortality worldwide, but unlike other task-shifting interventions, first aid has not been widely characterized or evaluated based on broad public health impacts.^2^ The connection between first aid education programmes and task shifting underscores how first aid interventions and lay emergency care might contribute to addressing priority global health challenges such as opioid poisoning, trauma or malaria.

We have summarized the breadth of contexts and conditions where lay responders, bystanders or friends and family can provide first aid. Leading international guidelines define first aid as “the initial care provided for an acute illness or injury” that “can be initiated by anyone in any situation.”^5^ The interventions and acute conditions included in this review conform with this definition, but many of the included studies concerned conditions and interventions that are mostly absent from conventional first aid training, such as lay assistance for acute malnutrition, opioid poisoning or paediatric communicable diseases. The appropriate scope of first aid and the set of interventions captured in those studies may be determined based on the care that can be initiated by anyone to provide initial care for an acute illness or injury in a safe and clinically effective manner. First aid need not be defined based on the set of interventions included in standard courses or curricula.

The strength of this review is its breadth, including a search of multiple databases and inclusive search terminology to synthesize the wide range of experimental and observational research concerning task shifting for emergency care in low-resource and underserved settings worldwide. By training and assessing interrater reliability of an international team of reviewers we were able to achieve a manual review of over 19 000 studies. Our approach to populations including both low- and middle-income countries and underserved subpopulations in high-income countries is a conceptual strength aligned with global approaches to health equity.^71^ Our review also has limitations. We excluded studies conducted in well-resourced populations because interventions that are effective in well-resourced settings cannot be presumed to work in contexts with fewer resources. For example, systematic reviews have demonstrated the efficacy of mental health first aid in high-income countries.^72^^,^^73^ Although mental health first aid has been studied in lower resource settings, we did not identify studies on mental health first aid reporting on an eligible health outcome in underserved populations. Our review uncovered only two studies reporting null results. This may reflect publication bias, though study heterogeneity prohibited testing of this hypothesis. The paucity of negative studies may reflect limitations in the methods of the included studies or that the interventions are broadly effective.

In conclusion, first aid for laypeople may have its greatest impact when approached as a series of targeted interventions that equip the public to respond to the health emergencies that they are likely to encounter in their everyday lives and communities. More work is needed to orient first aid education to deliver the greatest effects on patient and community health, and to identify the modalities that are best suited to specific contexts, populations, clinical conditions and public health priorities. Task shifting to laypeople for emergency care may save lives, reduce morbidity and enhance community capacity to address acute health problems in low-resource settings.
